# Cannabis and Its Potential Protective Role Against Inflammatory Bowel Disease: A Scoping Review

**DOI:** 10.7759/cureus.18841

**Published:** 2021-10-17

**Authors:** Nso Nso, Akwe Nyabera, Mahmoud Nassar, Mohsen S Alshamam, Vikram Sumbly, Mallorie Vest, Nehal Patel, Gilbert Ojong, Vincent Rizzo

**Affiliations:** 1 Internal Medicine, Icahn School of Medicine at Mount Sinai, Queens Hospital Center, New York, USA; 2 Internal Medicine, New York City Health and Hospitals/Queens, New York, USA; 3 Internal Medicine, Icahn School of Medicine at Mount Sinai, New York City Health and Hospitals/Queens, New York, USA; 4 Internal Medicine, Chicago Medical School, Chicago, USA; 5 Internal Medicine, La Magna Health/United Regional Hospital, Atlanta, USA

**Keywords:** cannabis science, ibd, internal medicine, preventative medicine, pain management

## Abstract

Globally, around 15%-40% of patients suffering from inflammatory bowel disease (IBD) use Cannabis for pain reduction, increased appetite, and reduced need for other medications. Although many patients report having benefited by using Cannabis in IBD, there is still a lack of consensus regarding the use of Cannabis in IBD. The aim is to identify, explore and map literature on the potential protective role of Cannabis against IBD through this scoping review. Preferred Reporting Items for Systematic Reviews and Meta-Analyses (PRISMA) guidelines were followed during the search to answer the focal question: (1) Does Cannabis play a protective role against IBD as assessed by clinical remission; (2) If yes, what is the mechanism of action for this protective role. There were only three randomized controlled trials (RCTs) and three observational studies that satisfied the selection criteria of this scoping review. Although promising results including the improvement in general well-being/ Harvey-Bradshaw Index, health perception enhancement [4.1±1.43 to 7±1.42 (p = 0.0002)], weight gain, Crohn's Disease Activity Index (CDAI) score<150, Mayo scores (4-10), and reduction in clinical complications have been found in some studies, its medical use in IBD is still questionable due to the lack of high-quality evidence. Future RCTs studies should determine the cannabis treatment parameters and validate its safety and effectiveness in the IBD setting. The highlights include: the current literature provides inconclusive evidence concerning the protective role of cannabis for IBD patients; limited research evidence regarding the therapeutic use of cannabinoids for IBD warrants future investigation via RCTs; cannabis provides some benefits to IBD patients by improving their general well-being perceptions, Harvey-Bradshaw Index, Mayo scores, and minimizing their clinical complications.

## Introduction and background

Inflammatory bowel disease (IBD) is a multifactorial gastrointestinal (GI) disease that causes chronic intestinal inflammation. Various predisposing factors of IBD include host genetics [1], uncontrolled inflammation due to dysregulated immune response to dietary antigens [2], exogenous factors such a4s western- diet based on high-fat dairy products, animal products, prepackaged food items, and processed meat [3], epithelial barrier defects [4] and gut-microbes [5]. These factors are found to play a crucial role in IBD. The major subtypes of IBD are Ulcerative colitis (UC) and Crohn's disease (CD). IBD usually presents as with symptoms such as abdominal pain, diarrhea, rectal bleeding, and weight loss [6] which is reported as an exaggerated response against microbial antigens. These changes cause structural damage of the intestinal mucosa in patients with a familial predisposition.

Various treatment modalities, including anti-inflammatories (aminosalicylic acid (5-ASA) and corticosteroids), immunosuppressants (azathioprine, and methotrexate) have been used to manage IBD. Biological agents such as antitumor necrosis factor (TNF)-a antibodies, vedolizumab (anti-a4b7 integrin antibody) and ustekinumab (interleukin [IL]-12/IL-23 antibody) have also been used to treat severe forms of IBD. Many of these treatment methods have been reported to have side effects such as opportunistic infections, malignancies, and infusion/injection reactions [7]. Hence, complementary and alternative medicine such as treatment with prebiotics, vitamins, probiotics, high-fiber diets, among others are becoming more popular. One such potential therapeutic choice is Cannabis sativa (CS) or its derivatives [8,9].

Cannabis is obtained from the Cannabis sativa plant. ∆9-tetrahydrocannabinol (THC) and cannabidiol (CBD) are among the most reported cannabinoids (active phytochemicals) that can activate endogenous Cannabinoid Receptors 1 (CB1) and 2 (CB2). The endocannabinoid system (ECS) contributes to gut hemostasis and comprises numerous endogenous receptors that regulate mucosal permeability and the gut microbiota that often triggers IBD following the loss of immunological tolerance. The ECS targets are the classic cannabinoid receptors (CB1 and CB2) and are known for its antinociceptive, anti-inflammatory, anti-diarrheal, antiemetics and analgesic effects. While Cannabis has been used vastly for various conditions such as rheumatic pain, constipation, its use has not been entirely legalized due to the tendency to develop a dependency, and cause abuse. Epidiolex® and Sativex® are among the first FDA-approved cannabis-based drugs to treat seizures, multiple sclerosis and cancer pain. Around 15-40% of patients [10], suffering from inflammatory bowel disease (IBD) use Cannabis for pain reduction, increasing appetite, and reducing the need for other medications [11]. Although many patients report having benefited by using Cannabis in IBD, there is still a lack of consensus regarding the use of Cannabis in IBD [12]. The medical literature does not specify any age-specific criteria for cannabis use for IBD due to the safety concerns based on a high risk of its potential abuse. Therefore, the main objective for the proposed scoping review is to identify, explore and map literature on the potential protective role of Cannabis against IBD as assessed by clinical remission.

## Review

Methodology

We included observational, retrospective and prospective studies, as well as randomized controlled trials (RCTs) published in English from January 2010 to December 2020. "Ulcerative Colitis" or "Crohn's Disease" or "Inflammatory Bowel Diseases" or "colitis"; and "Cannabis sativa" or 'cannabinoids" or "THC" or "cannabidiol" were the MeSH terms used in combination. PubMed/Medline, PMC, EMBASE, and Cochrane databases that were extensively searched.

The focal question used for this review was: (1) Does cannabis play a protective role against IBD as assessed by clinical remission; (2) If yes, what is the mechanism of action for this protective role". To answer these questions, the PRISMA guidelines were followed during the search and selection. A review protocol was prepared, but since this is not a systematic review, it was not registered in PROSPERO. E7ligibility criteria were decided before the commencement of the study. Study characteristics were identified based on population (patients belonging to any gender and age group with UC and CD who were treated with cannabis/cannabinoids); Intervention (cannabis/cannabinoids); Control (with or without placebo) and Outcome (improvement as measured by clinical remission).

Two hundred and sixty-four articles published between 2011 and 2019 were initially screened based on the study title. The screening included RCTs and observational studies; however, meta-analyses, systematic reviews, scoping reviews, editorials, and opinion papers were summarily excluded from the review process8,9. Fifty-four duplicate articles were removed by two independent reviewers while ensuring the inter-rater reliability. Thirty-five full-text articles were further screened to evaluate their applicability for this scoping review. Six studies were finally selected to analyze the protective role of cannabis against IBD.

Source Selection

Titles and abstracts obtained from the electronic bibliographic database search were screened for potential eligibility by two reviewers. Full texts were downloaded only for those studies which met the eligibility criteria. A training exercise was conducted for the reviewers and highly satisfactory (0.8 and above) kappa score was obtained to ascertain high inter-rater reliability. The guidelines given in the Cochrane handbook for systematic reviews were used to give direction to the discussion. Data was extracted from reports independently by two reviewers into an excel sheet. Disagreements between the reviewers were evaluated, discussed, and resolved by two other reviewers. The variables sought in these data included any mention of the gastro-protective role of Cannabis in cases of IBD.

The search results were screened independently by either of the two reviewers. Quality appraisal and risk of bias assessment was not done in this study as this is a scoping review. The aim was to map all available recommendations regarding the protective role of Cannabis against IBD. The principal summary measures included all details of the protective action of Cannabis in IBD. Since only a few RCTs of cannabinoids have been published, we included recent review articles on this topic to identify the research gap and map available literature.

Results

Study Objectives

Six studies were included. These were aimed at describing the effects of Cannabis in CD [13], assessing whether treatment with inhaled Cannabis improves the quality of life, reduces disease activity and promotes weight gain [14]. As well as determining whether Cannabis can induce disease remission in patients with Crohn's disease [15], the effects of cannabidiol on Crohn's disease [16], evaluation of efficacy, safety and tolerability of CBD-rich botanical extract in ulcerative colitis (UC) [17] and assessing the prevalence of complications arising due to Crohn's disease among cannabis users and non-users [18]. While some authors considered only one type of IBD, i.e., CD [15,16,18] or UC [17], Lahat et al. 2012 considered both UC and CD.

Age

The mean age of the participants ranged from 34±11.4 to 44±10.7. A total of 100 patients (68 males and 32 females) were recruited in three RCTs (a minimum of 19 and a maximum of 60 participants). Among the observational studies, the largest number of patients (n=615) were seen in a retrospective cohort study by Mbachi et al. and the least number of patients (n=13) were reported in the study by Lahat et al.

Type of Study

The included studies had different study designs. These were retrospective observational [13], open-label prospective single-arm trial [14], retrospective cohort study [18] and three randomized placebo-controlled trials [15,16,17]. All three RCTs were double-blinded. Only two studies [15,17] described the statistical power of the study as 80%, and only Naftali et al. 2017 mentioned the number of dropouts.

Disease Activity Index

The inclusion criteria of these studies were heterogeneous. Different disease activity indices for IBD were considered in these studies, such as Harvey-Bradshaw Index and general well-being-VAS [13], Harvey-Bradshaw Index; Partial Mayo Score based on the appearance of the intestinal mucosa [14], Crohn's Disease Activity Index (CDAI) scores >200 who did not respond to other treatment [15,16] and Mayo scores of 4-10 in endoscopy scores ≥1 [17]. Harvey Bradshaw Index is used for collecting clinical data related to CD using five clinical parameters, while Mayo score is to assess disease activity of UC based on bowel motions and presence of visible blood in it.

Cannabis Dose

Varying doses and forms of Cannabis were used in these studies. Cigarettes containing 50 gm dry-processed plant per month [14], cigarettes with 0.5 g of dried cannabis flowers equivalent to 11.5 mg THC [15], a low dose of CBD oil with10 mg of oil containing 5mg/ml [16] and hard gelatin capsules containing 50 mg CBD-rich botanical extract in excipients [17]

Mode of Administration

Most of these patients were prescribed cannabis inhalation/smoke [13,15], while others took it through water-bong [13] or orally [16, 17]. The average duration of treatment with Cannabis ranged from 8 weeks to 2.12 years.

Placebo

The placebo used in the three RCTs were cannabis flower content from which the THC had been extracted [15], olive oil [16] and capsules containing excipients only [17].

Primary Endpoint

There were many primary endpoints such as a reduction in disease activity and the reduced need for surgery [13], improvement in health perception, and (quality of life) QoL [14] induction of disease remission as measured by a CDAI score of 150 or less after eight weeks of cannabis treatment [15], CDAI reduction of 70 points from week 0 to week 8 [16], increase in the percentage of patients in remission after treatment [17] and reduction in active fistulizing disease and intra-abdominal abscess [18].

Secondary Endpoint

Statistically significant improvements in social functioning; ability to work; decreased bodily pain and depression; improved weight gain and BMI, and reduction in average Harvey-Bradshaw index [14], response rate as determined by a 100 point reduction of CDAI; reduction of at least 0.5 mg in CRP; and improvement in quality of life of at least 50 points, as measured by the Short form health survey (SF-36) [15], any adverse events within the treatment time frame of 8 weeks; ability to wean off steroids in patients, and reduction in at least 1 mg/dl in the CRP level [16], reduction in inflammatory marker levels such as blood C-reactive protein (CRP), plasma interleukin -2 and IL-6, tumor necrosis factor-alpha, and fecal calprotectin, inflammatory bowel disease questionnaire (IBDQ) score; physician global assessment of illness severity (PGAS) score; stool frequency and rectal bleeding on 4-point numerical rating scales (NRS); pain 0-10 NRS score; Mayo score and increase in body weight (Irving 2018), stricture complicating bowel disease; bowel obstruction; anemia; transfusion of blood products; parenteral nutrition requirement; small bowel resection; small bowel anastomosis, and partial or total colectomy [18].

QoL Improvement

Improvement in SF-36 Health Survey and EQ-5D Health Survey [14], a significant increase in quality of life as assessed by SF-36 [15], no withdrawal symptoms when stopping treatment at the end of the study [16] and patient-reported quality of life, as measured using the IBDQ and SGIC assessments improved in both groups but more in CBD group [17].

Adverse Effects

Only one study reported adverse effects in detail. More GI-related adverse effects, indicative of UC worsening, were seen in patients taking placebo [17]. Lahat et al. mentioned that patients did not report any adverse effects that affected their ability to work while using cannabis [14].

Smoking Cessation

Only Naftali et al. reported smoking cessation in some patients [13]. Of the 16 smokers who were included in the study, 3 stopped smoking before taking Cannabis. Among them, one showed significant improvement (Harvey Bradshaw score changed from 11-2), one showed slight improvement (HB score 9-7) and the last one showed no improvement (HB score of 4 before and after treatment). 

Statistical Analysis Used

Only patient characteristics and medical treatment list administered before and after the cannabis use were reported by Naftali et al. in the observational study [13]. Lahat et al. describe the changes in the SF-36v2 health survey, EQ-5D health survey, Harvey Bradshaw, CRP and BMI before and after cannabis treatment [14]. Descriptive and interferential analysis, including T-test, Chi-square, and the Fisher test, describe the demographic data, past and current medical treatment, and laboratory tests (Hemoglobin, HCT, WBC and CRP). Side effects were reported by Naftali et al. 2013 and Naftali et al. 2017 in their RCTs [15,16]. Both intention-to-treat (ITT) and per-protocol (PP) analysis were reported by Irving et al. in these RCTs [17]. 

In the study by Mbachis et al., Student's t-test, Wilcoxon rank-sum test, or Kruskal-Wallis test were used to compare the continuous variables in cannabis users with non-users while Chi-square test or Fisher's exact test was used to compare categorical variables [18]. Multivariable logistic regression analyses were done to evaluate clinical outcome as the dependent variable and cannabis use as the explanatory variable. A propensity score match was conducted by developing a model to predict cannabis use. The statistical assessments performed by the included studies signified the reliability of their results and helped minimize the risk of bias and confounded outcomes. The comprehensive assessment of these methods guided this scoping review and helped produce valid outcomes from the contemporary literature. 

Case Studies

The significant findings of these studies were the improvement in Harvey-Bradshaw Index and general wellbeing based on visual analogue scale (VAS) [13], statistically significant improvement in QoL and health perception (4.1±1.43 to 7±1.42 (p = 0.0002), disease activity and improvement in weight [14], complete remission in 5/11 cases with a CDAI score<150 [15], CDAI levels dropped both groups. NS [16], Patients were less tolerant of CBD-rich botanical extract compared with placebo [17] and the fact that cannabis users were less likely to have the active fistulizing disease and intraabdominal abscess; blood product transfusion; colectomy and parenteral nutrition requirement [18].

In the study by Naftali et al. 2011, all patients reported improvement in their general wellbeing. It was reflected by the positive change in the VAS score from 3.1 to 7.3 on a scale of 0 (very poor wellbeing) to 10 (excellent wellbeing) [13]. The Harvey-Bradshaw index (HBI) showed a drastic reduction from 14±6.7 to 7±4.7, and the average number of bowel movement reduced from 8 to 5 per day. An interesting phenomenon of steroid-sparing also was seen in this study, as only four patients required steroids at the end of the study instead of 26 in the beginning. 

Similar positive results were seen in the study by Lahat et al. [14]. Following treatment with cannabis cigarettes (50 g dry-processed plant per month), all the 13 patients included in the study perceived better general health, which was statistically significant (p = 0.001). Other parameters that showed simultaneous improvement was social functioning (p = 0.0002), ability to work (p = 0.0005), reduction in body pain (p = 0.004) and depression (p = 0.007). An average increase of 4.3±2 kg was observed during treatment (p = 0.0002), and an increase in BMI of 1.4 ±0.61 (p = 0.002) was also noticed. Reduction in the average Harvey-Bradshaw index (indicative of disease activity) from 11.36 ± 3.17 to 5.72±2.68 (p = 0.001) was also achieved. Inflammatory markers were recorded only in 6 patients. It was found that during treatment, the CRP levels returned to the normal range.

Naftali et al. found that when Cannabis was administered in the form of cigarettes containing 11.5 mg of tetrahydrocannabinol (THC), complete remission with a CDAI score of 100 was observed in 10/11 subjects in the cannabis group (90%; from 330±105 to 152±109) and 4/10 in the placebo group (40%; from 373±94 to 306±143; p = 0.028) which is indicative of disease remission. Weaning from steroid dependency (3/11), better appetite and sleep, with no significant side effects were reported in the cannabis group. Furthermore, a significant increase in quality of life, decreased physical pain, improved appetite and higher satisfaction from the treatment without any significant blood count changes, CRP or liver and kidney function were also reported. One interesting finding was that none of the patients complained of any withdrawal symptoms when cannabis use was discontinued at the end of the study [15].

However, Naftali et al. found that cannabinol (CBD) was safe but had no beneficial effect in managing moderately active Crohn's disease with a Crohn's disease activity index (CDAI) 200 or more. There was a decrease in the average CDAI following cannabidiol consumption from 337 ± 108 to 220 ± 122 in the CBD group and 308 ± 96 (p = NS) to 216 ± 121 in the placebo groups, respectively. No side effects or changes in the Hemoglobin, albumin, and kidney and liver function tests were observed [16].

Irving et al. found that patients on CBD-rich botanical extract were less tolerant than placebo and showed less compliance with the protocol. A subjective evaluation showed more significant improvement in the CBD group regarding patient-reported quality-of-life outcomes (p = 0.065 and overall improvement (p = 0.003). Adverse events (AEs) were reported in both groups, with a more significant proportion of GI-related adverse effects in the placebo group indicative of UC worsening, probably due to the ∆9 -tetrahydrocannabinol content. Mild to moderate dizziness and somnolence were the most common treatment-related AE and was observed more in patients taking CBD-rich botanical extract. However, adverse effects of the abdominal area were found to be more magnified in the control group (42%) than in those in the test group (10%) [17]. 

Mbachi et al. show an association between the use of Cannabis and improvement in many of the commonly documented complications of CD, such as active fistulizing disease and intraabdominal abscess, blood product transfusion, colectomy, and parenteral nutrition requirement [18]. The results of this study are interpreted, taking into account the many confounding factors that could be present (Tables 1, 2).

**Table 1:** Characteristics of the included study. CD: Crohn's disease; UC: ulcerative colitis; CDAI: Crohn's disease activity index; THC: tetrahydrocannabinol.

Sl no	Author and year	Aim	Type of IBD	Mean age (years)	Sample size (M/F)	Disease activity Index	CBD dose	Mode of administration	Placebo	Treatment duration	Results
1	Naftali et al. 2011 [13]	Elaborate effects of Cannabis in CD	CD	36	30 (26/4)	Harvey-Bradshaw Index; General wellbeing index using-VAS	Varying	Most patients smoked; through water-bong; one patient- orally	Not applicable	The average duration of Cannabis 2.12 years	Improvement in Harvey-Bradshaw Index and general well-being.
2	Lahat et al. 2012 [14]	Effect of inhaled Cannabis on quality of life, disease activity and weight gain.	Long standing IBD	44±10.7 (CD); 29.5±2.1 (UC)	13 (9/4)	Harvey-Bradshaw Index; Partial Mayo Score- the appearance of the mucosa	50 gm dry cannabis per month when they felt pain; up to 3 inhalations.	Inhaled Cannabis; cigarettes	No control group	Three months	Improvement in quality of life, disease activity and weight gain.
3	Naftali et al. 2013 [15]	Can Cannabis induce remission in patients with CD?	CD	40±14	21 (13/8)	CDAI scores >200 who did not respond to other treatment.	Cigarette with 0.5 g of dried cannabis flowers (=11.5 mg THC)	Smoke	Content of the cannabis flower from which THC had been extracted	During eight weeks of treatment and two weeks thereafter.	Complete remission 5/11 with a CDAI score<150.
4	Naftali et al. 2017 [16]	Effects of cannabidiol on Crohn's disease	Moderately active CD	39±15 (CD)	19(11/8) other medical illness	CDAI >200	CBD oil (5mg/ml) 10 mg Oral (Low dose) (n=10/9)	Oral	Olive oil	Eight weeks	CDAI levels dropped both groups; statistically non-significant.
5	Irving et al. 2018 [17]	Efficacy, safety and tolerability of CBD-rich botanical extract in UC	UC	18 yrs or older	60 (44/16)	Mayo scores of 4–10; (endoscopy scores ≥1)	Hard gelatin capsules containing 50 mg CBD-rich botanical extract in excipients (n=29/31) Cannabidiol 4.7% THC	Oral	capsules containing excipients only.	Ten weeks	Patients were less tolerant of CBD-rich botanical extract compared with placebo.
6	Macchi et al. 2019 [18]	To compare the prevalence of CD related complications among cannabis users and non-users	CD	34±11.4	615	Not mentioned	Not mentioned	Not mentioned	Not applicable	Not applicable	Cannabis users were less likely to have the active fistulizing disease and intraabdominal abscess, blood product transfusion, colectomy and parenteral nutrition requirement.

**Table 2:** Characteristics of the included study (contd.). SF-36 Health Survey: Short form health survey; EQ-5D Health Survey: EuroQol- 5 dimension health survey; CRP: C-reactive protein; IL: Interleukin; TNF: Tumor necrotic factor; IBDQ score; Inflammatory bowel disease questionnaire score; PGAS score: Physician global assessment scores; NRS: Numerical rating scale; ITT: Intention to treat analysis;  PP: per-protocol analysis.

Sl no	Author and year	Type of study	Blinding	Power of study	Dropouts	Primary Endpoint	Secondary Endpoint	QoL improvement	Adverse Effects	Smoking cessation	Analysis done
1	Naftali et al. 2011 [13]	Retrospective observational	Not applicable	Not applicable	Not applicable	Reduction in disease activity, need to surgery reduced	Not applicable	Not mentioned	None reported	Yes	Descriptive
2	Lahat et al. 2012 [14]	Open-label, prospective, single-arm trial.	Not applicable	Not applicable	Not applicable	Improvement in health perception 4.1±1.43 to 7±1.42 (p = 0.0002).	Statistically significant improvement in social functioning; ability to work; physical pain and depression; 4.3±2 kg during treatment; and an average rise in BMI of 1.4 ± 0.61. The average Harvey-Bradshaw index was reduced from 11.36 ±3.17 to 5.72± 2.68	SF-36 Health Survey EQ-5D Health Survey	No significant adverse effects reported	No	Correlational analysis
3	Naftali et al. 2013 [15]	RCT	Double-blinded	80%	Not mentioned	Induction of remission, defined as a CDAI score of 150 or less after eight weeks of cannabis treatment.	Response rate, determined as a 100 point reduction of CDAI, reduction of at least 0.5 mg in CRP or improvement in the quality of life of at least 50 points, as measured by the Short-form health survey (SF-36)	Significant increase in quality of life as assessed by SF-36	Sleepiness, nausea, mild memory loss, dizziness (only in a few		T-test, Chi-square, Fisher test
4	Naftali et al. 2017 [16]	RCT (block)	Double-blinded	Not mentioned	One	CDAI reduction of 70 points from week 0 to week 8.	Any adverse events within the time frame of 8 weeks, the ability to stop steroids in patients treated with steroids at the beginning of the study, and reduction in at least 1 mg/dl in the CRP level.	QoL was taken but not mentioned in the result	Headache, sleepiness, nausea, dizziness; statistically not significant.	No	T-test, Chi-square, Fisher test
5	Irving et al. 2018 [17]	RCT	Double-blinded	80%	Not mentioned	Percentage of patients in remission after treatment	Inflammatory marker levels (blood CRP, plasma IL-2, IL-6, TNF-α, and faecal calprotectin; IBDQ score; PGAS score; stool frequency and NRS; Mayo score; body weight	Patient-reported quality of life, as measured using the IBDQ and SGIC assessments, improved in both groups but more in the CBD group	Gastro-intestinal-related adverse effects, indicative of UC worsening	Not mentioned	Both ITT and PP
6	Macchi et al. 2019 [18]	A Retrospective Cohort Study	Not applicable	Not applicable	Not applicable	Active fistulizing disease and intra-abdominal abscess,	Bowel obstruction, anemia, transfusion of blood products, parenteral nutrition requirement, small bowel resection, small bowel anastomosis, and partial or total colectomy	Not applicable	Not mentioned	Not mentioned	Multivariable logistic regression

Endocannabinoids

Some of the natural cannabis compounds used for IBD are Cannabidiol (CBD), cannabigerol and cannabichromene. CBD is known to have anti-inflammatory properties without adverse psychotic effects [16]. Cannabis and its constituents act through CB1 and CB2 receptors situated in different GI system areas such as the liver, pancreas, stomach, small and large intestines. Activation of these CB1 and CB2 receptors produce antiemetic, anti-motility, and anti-inflammatory effects through inhibition of adenylyl cyclase with the reduced cAMP formation, which inhibits neurotransmitter release from a presynaptic neuron by CB1 and pro-inflammatory cytokine release by CB2 [19,20]. When cannabinoids bind to the prejunctional CB1 receptors, decreased gut motility and secretion occurs due to reduced excitatory neurotransmission causes [21]. Research shows that activation of CB1 receptors also helps control emesis, reduces peripheral inflammatory hypersensitivity and hyperalgesia, along with the control of centrally originating pain.

The Dependency of Cannabis in OBD

Dependency and potential for abuse are among the common problems associated with chronic cannabis users due to impairment of mental and cognitive functions. The dependency also affects the heart, lungs and psyche. Both observational and placebo-controlled studies of Cannabis in IBD did not show any substantial abuse potential, possibly because of their smaller sample size and short duration of treatment [15]. Prevalence of cannabis abuse is more in the general population than patients with IBD complaints and similarly in IBD patients compared to controls [22]. Weiss et al. reported more use of Cannabis in control subjects than patients with IBD (72.9% vs 63%), which could impose a higher risk of cannabis dependency in IBD patients [23]. Synergistic cannabinoids were demonstrated in a study by producing better anti-hyperalgesia through vanilloid TRPV1 receptors [24].

Protective Effects of Cannabis against IBD

There were three RCT and three observational studies included in this scoping review. The efficacy and protective role of Cannabis can be evaluated only through RCTs. However, this scoping review does not elaborate on the studies' risk bias, data extraction high-risk bias in most of these studies. Case definitions for IBD varied in the study. Also, we found that Naftali et al. [15] had older patients in the cannabis group than the placebo group participants. Moreover, despite the randomization of participants, several participants could have found out their treatment group due to the psychotropic effects of Cannabis [9]. In the study by Naftali in 2017 [16], more than half of the cannabis participants were smokers compared with none of the placebo participants. It should be noted that smoking is contraindicated in IBD, particularly CD, and so it would not be appropriate to advocate a smoked product. Among these, the overall risk of bias was low for the study by Irving et al. [17]. A proper sample size calculation based on the study's power done a priori is an essential step in any clinical trial. Among the included study only Naftali et al; and Irving et al. described the study's statistical power as 80%, but none reported the formula used [15,17].

There is no direct evidence regarding the best protective dose of Cannabis in IBD. Patients who used Cigarettes containing 50 gm dry-processed plant per month [14], did not report any adverse effects that disturbed their working ability using Cannabis. In another study by Naftali et al., although induction of CD remission was not achieved with cigarettes containing 0.5 g of dried cannabis flowers equivalent to 11.5 mg THC, they concluded that THC produced a significant clinical steroid-free benefit in all but one active CD patient [15].

However, another randomized, placebo-controlled trial by Naftali et al., oral (sublingual oil droplets) 10 mg twice daily dose of CBD in 20 moderately active CD showed no beneficial effects [17]. This could be attributed to the low doses of CBD or a lack of synergism with other cannabinoids. Recently, a Cochrane review [9] did not find any conclusive results regarding Cannabis's beneficiary role in clinical remission of CD, which could again be due to small sample sizes in three RCTs.

Two main statistical approaches used to analyze RCT data are the intention to treat (ITT) and per-protocol (PP) analysis. At the same time, ITT analysis measures the effect of assigning a treatment, PP analysis measures receiving a treatment. Irving et al. used both these to interpret the results of their RCTs [17]. In ITT analysis, participants who drop out of the study or are not compliant to study medications are also considered in the trial's primary analysis. This method's advantages include retaining the balancing of the risk factors between the study arms at baseline due to randomization and keeping the study power unchanged as no patient was removed from the analysis [25]. PP analysis is a complementary method to investigate the actual effect of administered treatment strategies throughout the follow‐up period. Hence, it only includes the subgroup of patients who completed the RCT trial without significant deviations from the proposed protocol. Patients who dropped out switched the allocation arm of treatment or did not participate for re-evaluation represent the "true" exposure to treatment.

Another proposed model of the protective action of Cannabis is by the activation of the Cannabinoid receptor on hematopoietic cells and enterocytes, as seen in experiments on female C57BL/6 mice [26]. The authors report that THC prevented colitis while CBD offered no significant change in reducing colitis. THC was found to enhance colonic barrier integrity of the large intestine by activating mucus, tight junction and antimicrobial peptide production, and an increase in colonic gram-negative bacteria. Anti-colitic effects of THC were independent of the microbiome. THC acted on both immune cells via CB2 and on enterocytes to attenuate colitis.

The broad activity spectrum of CBD includes antioxidant and anti-inflammatory activity as it is associated with improvement in redox imbalance and inflammation. Evidence shows that CBD reduces the levels of pro-inflammatory cytokines, prevents T cell proliferation, induces T cell apoptosis and decreases migration and adhesion of immune cells [27]. In addition, CBD anti-inflammatory activity has been found to be antagonized by both a selective CB2 antagonist and AEA, an endogenous CB2 receptor agonist [28].

In the final analysis, the overall results of the study should be interpreted considering the subjective nature of many parameters and the psychedelic nature of Cannabis. Moreover, the general well-being reported in the patients could be attributed to the symptomatic relief of gastrointestinal-related issues. Since endoscopic healing data and objective inflammatory markers from these patients are not available, there is no actual evidence that indicates that Cannabis modified the disease. Further research is needed to ascertain that Cannabis plays a protective role by modulating the disease and not just alleviating IBD symptoms.

Future perspective

Cannabis is still considered a Schedule I drug with high abuse potential and non-usable as medicine by the FDA and the Drug Enforcement Agency (DEA). Even if the initial results show some favourable results, the legal and social implications of the unmonitored use of Cannabis should be allowed only with caution, despite its benefits. Despite reports of improvement in QoL and amelioration of gastrointestinal symptoms, there is no scientific evidence that proves the ability of Cannabis to modify disease objectively. It will be possible by recording the biomarker profiles and endoscopic healing of these patients [29]. There is a need for more in vitro and in vivo studies, and more extensive trials with proper randomization are needed to have a complete understanding of the protective role of Cannabis in IBD. In this scoping review, we have tried to discuss the protective effects of Cannabis in IBD comprehensively [11]. Although promising results including the improvement in general well-being/ Harvey-Bradshaw Index, health perception enhancement [4.1±1.43 to 7±1.42 (p = 0.0002)], weight gain, CDAI score<150, Mayo scores (4-10), and reduction in clinical complications have been found in some studies, its medical use in IBD is still questionable due to the lack of high-quality evidence-based studies. There is also a need to design safer cannabinoid derivatives for use via randomized clinical trials with a larger sample size. This is necessary for determining the appropriate dose, mode of administration, protective effects and side effects of Cannabis before it can be accepted as a possible therapeutic agent for IBD.

Limitations

First, the high heterogeneity in the findings from the included studies restricted the generalizability of the outcomes from this scoping review. This heterogeneity was reflected in case definition, inclusion criteria, cannabis dosage, mode of administration of Cannabis or cannabinoid, duration of treatment, disease activity indices, and study design of the included studies. Second, the small sample size further reduced the validity and reliability of the reported results. Third, the limited number of RCTs and inclusion of observational studies increased the risk of selection and detection biases that further reduced the strength of our findings.

## Conclusions

This scoping review shows inconclusive results regarding the benefits and harms of Cannabis and cannabidiol in patients with active CD or UC. Only a few studies report the management of active CD and UC with Cannabis or cannabinoids but these lack follow-up data. The findings, however, confirm the improvement in general well-being/Harvey-Bradshaw Index, weight gain, and reduction in clinical complications after cannabis treatment. Future RCTs studies should determine the cannabis treatment parameters and validate its safety and effectiveness in the IBD setting. 
